# Unraveling how the third-party brain under stress responds to injustices

**DOI:** 10.1371/journal.pbio.3002618

**Published:** 2024-05-17

**Authors:** Masahiko Haruno

**Affiliations:** 1 Center for Information and Neural Networks, NICT, Osaka, Japan; 2 Graduate School of Frontier Biosciences, Osaka University, Osaka, Japan

## Abstract

How third-party individuals respond to injustices is important for resolving conflict in society. This Primer explores a new study that shows that individuals experiencing acute stress prefer to aid victims over punishing offenders, an opposite pattern to non-stress conditions.

## Teaser

How third-party individuals respond to injustices is important for resolving conflict in society. A study in *PLOS Biology* shows that individuals experiencing acute stress prefer to aid victims over punishing offenders, an opposite pattern to non-stress conditions.

The response of third parties, who are not directly involved in injustices, has a crucial role in restoring justice [1]. This principle holds true across various aspects of our society, from daily familial issues to international conflicts. Previous research has demonstrated that people typically prefer to punish offenders rather than help victims [2]. This inclination suggests a natural (default) human bias towards justice, aiming to correct wrongs by penalizing the wrongdoer. However, in such antisocial contexts, third parties are also under stress, and the impact of this stress on third-party punishment and assistance remains largely unexplored. A new study by Wang and colleagues [3] offers fresh insights into how acute stress influences third-party responses to antisocial behavior, revealing that the default option shifts significantly when the intervenor is under acute stress.

In exploring the nexus between stress and intervention strategies, the study employs a sophisticated blend of the cold pressure test [4] and a computational model-based functional magnetic resonance imaging (fMRI) within a novel third-party intervention game [3]. This methodological innovation allows for a precise examination of how acute stress alters the decision-making process in scenarios of unfairness. The main game tasks third-party participants with deciding how to allocate their monetary units (MUs) after observing 2 other individuals interacting with one another. The interactions occur within the context of another separate game that the 2 other individuals play with one another. Specifically, in each trial of this second task, one of the individuals (the proposer) proposes a way to share an additional 100 MUs with the other individual (the receiver). The proposals could be fair or unfair. The third-party participants are then asked to choose from 3 behavioral options within 4 s: subtract from the proposer, add to the receiver, or keep the MUs using their own endowment of 50 MUs during the decision phase. Subsequently, they decide how many MUs to deduct from the proposer or to grant to the receiver (transfer phase).

Behaviors indicated that acute stress decreases third parties’ willingness to punish norm violators and the severity of the punishment, but increases their willingness to help victims in extremely unfair conditions. Computational modeling separated the latent computations for the severity of punishment (i.e., how averse a person is to observe someone else hurt others) and the extent of help (i.e., how averse a person is to observe someone else being hurt) by introducing advantageous and disadvantageous inequity between the proposer and the receiver. Crucially, the difference between coefficients for the 2 terms accurately captured individual punishment bias.

In the fMRI analysis of the choice phase, the stress group exhibited higher activation in the left amygdala and bilateral insula compared to the control group. In the stress condition, the right amygdala showed lower correlation with inequity than in the control condition. The authors also found that the functional connectivity between the right amygdala and the ventromedial prefrontal cortex (vmPFC) was higher when participants punished transgressors than when they helped victims. Consistently, higher activity in the right dorsolateral prefrontal cortex (DLPFC), anterior cingulate cortex (ACC), and posterior cingulate cortex (PCC) during stress than in the control condition was observed only during punishment choices.

In line with this, the authors also observed a similar activity pattern in the transfer phase: the severity of punishment (i.e., the difference between the 2 inequity coefficients in the utility function) and activity in the right ACC and right PCC correlated in opposite directions (positive and negative) in the stress and control conditions. These findings suggest that these 2 cortical structures are involved in the process of reversing a default behavioral option.

The study by Wang and colleagues [3] revealed neurocognitive mechanisms for acute stress-induced changes in third-party help and punishment, making significant contributions to our understanding of human prosocial behavior. Simultaneously, this study suggests many intriguing questions for future research. For instance, the brain computation mechanism for selecting a default option, that is, transforming “inequity” into helping victims and punishing transgressors in stress and control conditions, remains unaddressed. The emotional salience network, including the amygdala and insula, is likely involved. Particularly, considering the authors’ observation that the right amygdala showed decreased correlation with “inequity” under stress, the amygdala may hold a central role in the default choice, consistent with previous studies on its involvement in prosocial behavior based on inequity aversion [5–7]. Moreover, acute stress does not necessarily promote prosocial behavior in real society; it can also enhance antisocial behaviors, such as ignoring or even conforming to the transgressor [8]. Investigating third-party antisocial behaviors under acute stress may also be an important direction.

In conclusion, the study by Wang and colleagues [3] not only elucidates the acute stress-induced shift in third-party intervention strategies but also highlights the nuanced and multifaceted nature of human social behavior under stress. As this research unfolds, it promises to deepen our comprehension of the social and neurocognitive underpinnings of prosocial and antisocial behaviors, offering valuable insights for addressing conflicts and promoting a more empathetic society.
